# Computational Study of Stress Distribution in Polyethylene Elements Due to Metal Components of Knee and Hip Implants Made from Different Metal Alloys

**DOI:** 10.3390/ma18163924

**Published:** 2025-08-21

**Authors:** Michał Sobociński, Marcin Nabrdalik

**Affiliations:** Department of Technology and Automation, Faculty of Mechanical Engineering, Czestochowa University of Technology, 42-201 Czestochowa, Poland; marcin.nabrdalik@pcz.pl

**Keywords:** polymer composites, polyethylene ultra high molecular weight, finite element method, ceramics

## Abstract

The complexity of the processes occurring in both natural and artificial joints necessitates carrying out the analysis on a 3D model based on already existing mathematical models. All the presented numerical calculations define qualitative conclusions about the influence of certain parameters of endoprostheses on the values of stresses and strains arising in polyethylene parts of hip and knee endoprostheses. The obtained results make it possible to reveal “weak points” in the studied models and thus counteract the later effects resulting from premature wear of the endoprosthesis components. The study included a numerical analysis of the stress and strain distribution of polyethylene components of hip and knee endoprostheses working with the most commonly used material associations in this type of solution. The most common are metal alloys and ceramics. The analyses were carried out using ADINA and Autodesk Simulation Mechanical software. Geometric models were designed based on current solutions used by leading endoprosthesis manufacturers. The load models adopted are based on models commonly used in musculoskeletal biomechanics. Particular attention was paid to modeling the resistance due to friction at the hip endoprosthesis node. To build the hip endoprosthesis model, eight-node 3D solid elements were used. Due to the axisymmetric geometry of the model, the resulting discrete model consisted of 10,000 cubic elements described by 10,292 nodes. In the case of the knee endoprosthesis, a finite element mesh was adopted for the calculations, which was built with 3600 3D solid cubic elements and 4312 nodes. The accuracy of the adopted numerical model did not differ from the generally used solutions in this field.

## 1. Introduction

In the group of materials used for hip and knee endoprostheses, polyethylene is an important item. One of the main problems related to the production and use of plastics in implantology is the pursuit of a material that, with its chemical and phase composition, structure and resulting properties, is a substitute for bone tissue that has been worn out or removed as a result of destructive processes. An important aspect is obtaining high mechanical strength, a low coefficient of friction in cooperation with other elements of the endoprosthesis. It is also about maintaining tribological properties during the process of use in difficult “in vivo” conditions.

Ultra high molecular weight polyethylene, polyamide and polyethylene terephthalate are different types of plastics, each with unique properties and applications. UHMWPE (ultra-high molecular weight polyethylene) is characterized by exceptional tribological wear resistance and impact strength, but difficulty in processing. PA (polyamide, such as nylon) is distinguished by its high strength, flexibility and abrasion resistance, but it can be prone to moisture absorption. PET (polyethylene terephthalate) is a popular packaging material with good gas barrier and transparency, but it may have lower resistance to high temperature and abrasive wear.

UHMWPE is used for endoprosthesis components due to its unique plastics properties such as very high abrasion and impact strength, low coefficient of friction, good chemical resistance and biocompatibility. Coatings are applied to the surfaces of polyethylene endoprosthesis components, which, in the friction node part, reduce the wear of the component, while on the outer side, for example, the acetabulum of a hip endoprosthesis, they promote stabilization by improving the overgrowth of bone tissue into the implant.

The application of modular endoprosthetic systems facilitates the precise selection of friction pairings that correspond to the patient’s specific biomechanical requirements. Furthermore, such systems enable the isolated replacement of worn or damaged articulating components, thereby minimizing the extent of potential revision procedures and preserving surrounding anatomical structures.

At the initial stage of endoprosthesis design, analytical solutions should be used to indicate areas where damage or premature wear of cooperating elements may occur [1,2]. Most mechanical damage in alloplasty results from material fatigue. All numerical calculations described in the literature allow for drawing valuable conclusions regarding the influence of selected parameters of use on the values of stresses and strains in individual elements of the endoprosthesis [3,4]. Based on the conducted analyses, it was found that the weakest element of the endoprosthesis is the polyethylene insert [5,6]. The presented numerical analysis is the fastest and clearest method for defining the distribution of stresses and strains occurring in the elements of joint endoprosthesis.

The ceramic insert of endoprostheses, unlike the metal one, is characterized by less wear and higher scratch resistance. However, its main disadvantage is its fragility and risk of fracture. In clinical implications, the ceramic head of a hip endoprosthesis provides long-term performance but requires care in selection and use to avoid complications.

As for the clinical implications of the ceramic head in hip endoprostheses, the following advantages can be pointed out:

(1) Less wear and tear: Ceramic is a more abrasion-resistant material than metal, resulting in longer endoprosthesis life.

(2) Lower risk of allergic reactions: Ceramic is a biologically inert material, which minimizes the risk of allergic reactions in patients.

(3) High biocompatibility: Ceramics do not cause negative reactions when in contact with tissues, which promotes healing and integration of the implant with bones.

Unfortunately, ceramic components also have disadvantages, the biggest of which are the following:

(1) Fragility and risk of fracture: Ceramic, despite its advantages, is a brittle material and can fracture as a result of trauma or excessive loading.

(2) Potential risk of ceramic particle release: In the event of fracture, fine ceramic particles can be released, which can lead to an inflammatory reaction in the joint.

(3) Limitations on patient selection: Due to their fragility, ceramic heads are not recommended for all patients, especially those who are highly active or overweight.

The friction and lubrication phenomena in the implanted joint are significantly different from the natural one. The materials from which the endoprosthesis is made have different properties than bone tissue. Above all, there is no flexible articular cartilage covering the bony elements that work together. In a joint with an implanted prosthesis, there is a much higher frictional resistance than in a healthy joint. It is important that the endoprosthesis has the correct geometry; otherwise, the frictional resistance will take on higher values and the materials will wear out much more quickly.

Currently, a widely proposed solution for the construction of endoprostheses by manufacturers is the use of a replaceable cup in a metal housing. This solution reduces the extent of the revision surgery that the patient must undergo after the wear of the friction elements of the endoprosthesis. Cups of this solution usually consist of a metal housing made of the Ti6Al4V titanium alloy and an appropriate insert. The replaceable internal element can be made of UHMWPE [7,8,9], bioceramics or two-layer materials.

The finite element method (FEM), despite its popularity, has several important limitations. First of all, it is a numerical method, which means that the results are approximate and subject to errors. The main limitations are modeling errors, coefficients, area mapping, numerical errors leading to discretization errors and rounding errors. As for modeling errors, these mean that the mathematical model used in the FEM may not reflect reality very perfectly. This applies to both the geometry of the model under study and the behavior of the material of which it is built. Another error is the error of coefficients. The values of coefficients in equations (e.g., material parameters and boundary conditions) are often estimated and subject to error.

Additionally, an important error is the so-called area mapping error, which is that the finite element mesh does not always perfectly represent the actual analysis area, which can introduce additional errors.

The numerical error mentioned earlier means that the discretization of the area into finite elements introduces an error compared to the exact solution of a continuous problem.

Finally, rounding errors are related to the limited precision of computer calculations (e.g., floating-point numbers).

## 2. Materials and Experimental Procedure

### Materials and Models

The model assumes the dimensions of an actual modular acetabulum and an endoprosthesis head from one of the world’s endoprosthesis manufacturers.

The geometric model was developed based on measurements of actual cups and heads and on the dimensions provided by the manufacturer in the product catalog [10]. The solid model was built directly in the ADINA program. Due to the difficulties caused by modeling the “cup housing-bone” connection, the contact was simplified to a common edge. Technological holes used to secure the cup during the implantation procedure, causing stress concentration in the observed model, were also omitted.

Two engineering software programs, SolidWorks and ADINA [11,12], were used to build the endoprosthesis knee model and conduct the study. SOLIDWORKS^®^ was used to create the geometric model of the sled and the polyethylene insert. Using ADINA, a solid model and a discrete model, calculations using the finite element method were created, and the results obtained were presented.

The material model of the “endoprosthesis head-acetabulum” system was developed based on the results of experimental studies available in the literature of the mechanical properties of bone structures and data on the mechanical properties of implant materials [13,14,15,16]. The anisotropy of mechanical properties and viscoelasticity of bone tissue were omitted in the simulations.

In all cases, bone tissue was assumed as an elastic isotropic material. The following types of materials used for the “head-acetabulum” nodes of the hip joint endoprosthesis were included in the calculation models: Ti6Al4V titanium alloy, CoCrMo alloy and Al_2_O_3_ ceramics. The strength parameters of materials and bone tissue adopted for calculations are listed in Table 1.

**Figure 1:**
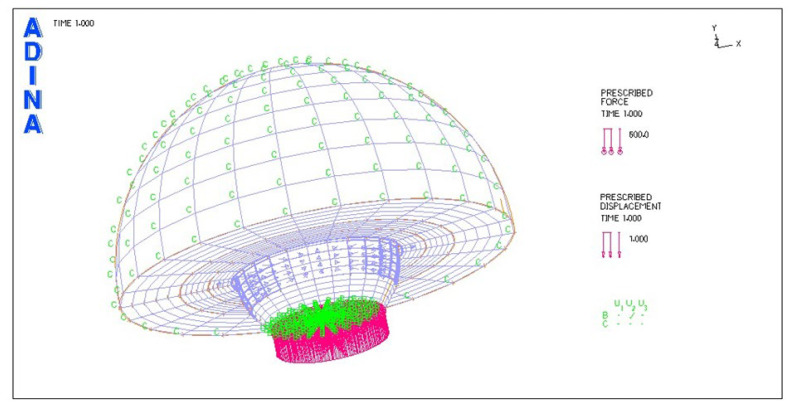
Discrete model view of the modeled solution of hip joint endoprosthesis.

Figure 1 illustrates a discrete model view of the hip endoprosthesis.

As part of the work, three discrete models of the “endoprosthesis head-cup” system were developed for four material associations:

CoCrMo head-UHMWPE cup;

Ceramic head-UHMWPE cup;

Ceramic head-ceramic cup.

In the tested system, a load system corresponding to the force loading the endoprosthesis head in the XY plane with a force of 600 N and 1500 N was adopted.

To obtain stability of the system under consideration, the tested model was rigidly fixed in the external plane, i.e., on the surface corresponding to the external part of the bone tissue surrounding the implant, by taking away all degrees of freedom from the nodes lying in the fixation plane [17,18,19,20,21,22,23,24,25,26,27,28,29,30,31,32].

**Figure 2:**
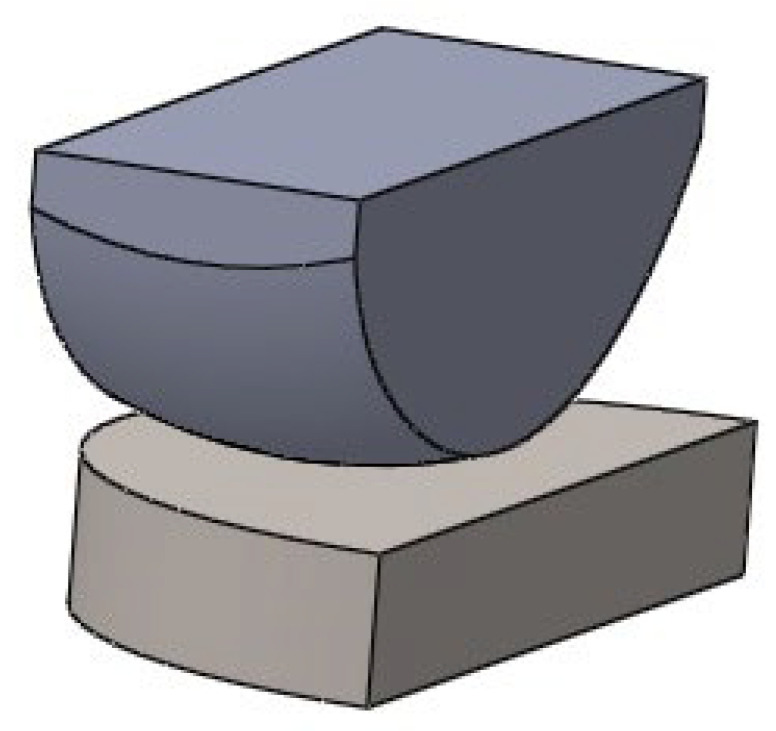
Simplified sled and insert model.

This is the ENDO-MODEL single-articular sled endoprosthesis model from W. LINK. This implant is indicated for patients with mild damage to the articular surfaces of the bone and an intact ligamentous apparatus. During the creation of the model, elements of the endoprosthesis that do not directly affect the calculation results were simplified. In the model, the fixation pins and the inner surface of the sled in contact with the femoral condyle surface were simplified. The overall shape of the implant was obtained by “pulling” solids from previously created individual sketches of the sled and insert. The 3D model created is shown in Figure 2.

**Figure 3:**
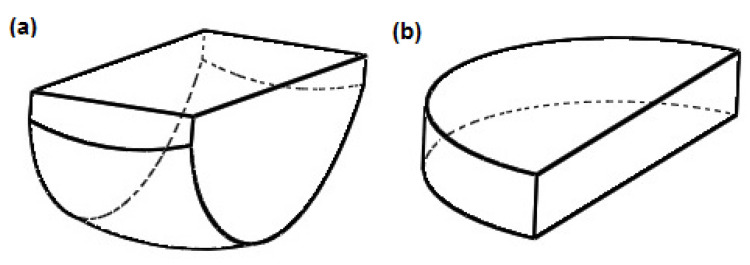
Edge representation of the endoprosthesis components: (**a**) sled and (**b**) insert.

The endoprosthesis model shown is a solid-surface model. The sled is a solid consisting of 6 surfaces, while the insert consists of 4 surfaces. The edge models of the sled and the insert are shown in Figure 3a,b.

**Figure 4:**
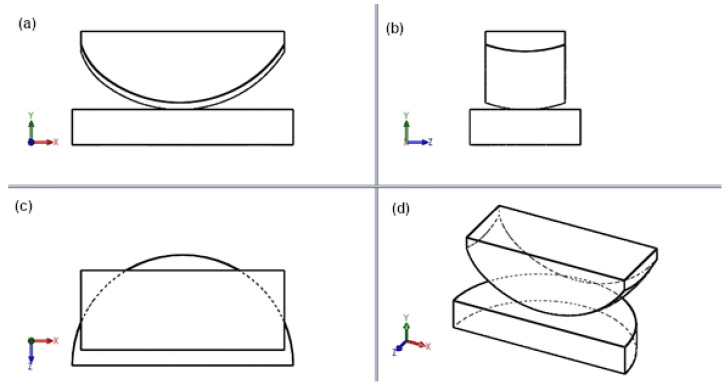
Model of endoprosthesis in four projections—edge representation: (**a**) side view, (**b**) front view, (**c**) top view, (**d**) axonometric projection.

Figure 4 illustrates a 3D edge model of a sled endoprosthesis in four projections, created in SolidWorks.

**Figure 5:**
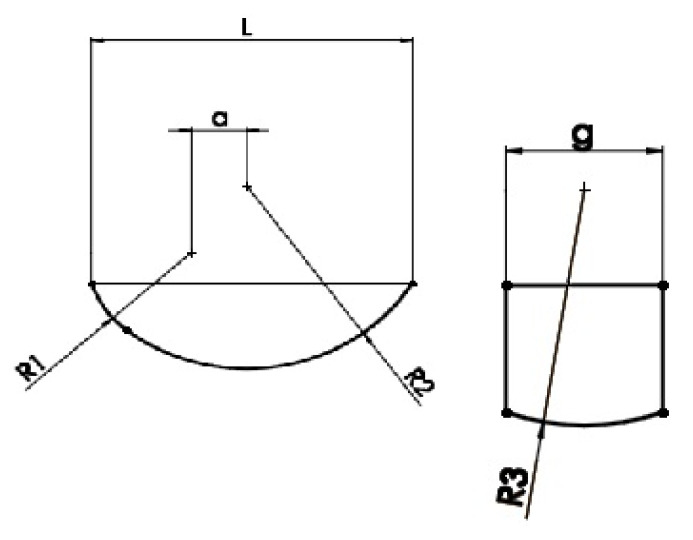
Basic dimensions of the simplified sled model.

**Figure 6:**
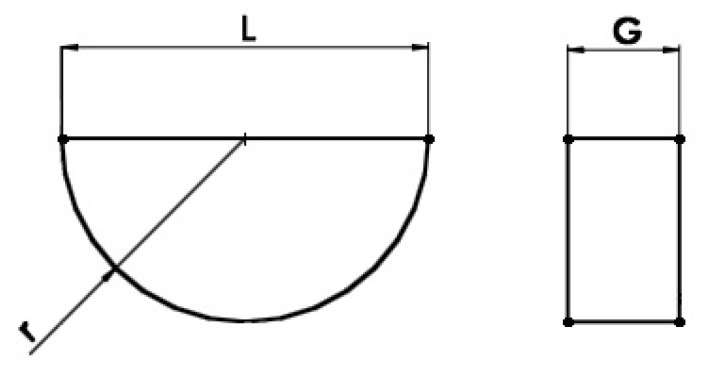
Basic dimensions of the simplified insert model.

The basic dimensions of the sled (Figure 5) and the insert (Figure 6) are shown below.

The models will consist of a combination of three sleds and three inserts, differing in geometry and material type. The variable value for the sled model is the cross-sectional radius R3, while the thickness, denoted by the symbol G, will vary in the insert. The remaining geometric values are constant for all sled and insert models. The geometric dimensions of these endoprosthesis components are listed in Table 2 and Table 3.

The combination of all of the above produces nine models on which the calculations will be carried out. The designations of the individual models are shown in Table 4.

In addition, each model will be loaded with a normal force of values: 800 N, 1200 N and 2000 N.

**Figure 7:**
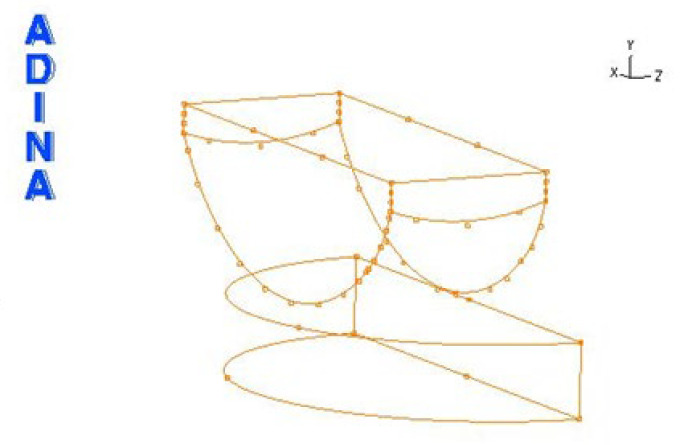
Model geometry imported into ADINA.

The creation of the model started with importing a geometrical model from SolidWorks, saved in IGES format. In this format, since the model is composed of points and lines, it is necessary to create surfaces and volumes. The process of creating the model is illustrated using an R27G8 model loaded with a force of 2000 N. Figure 7 shows the model after importing the file into ADINA.

**Figure 8:**
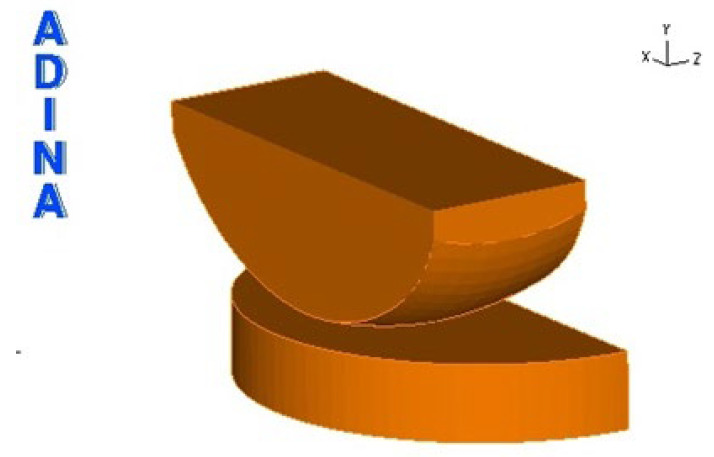
Endoprosthesis model after volume creation.

The next step was to create the surface and then the volume of the skid and the insert. After these steps, the model looks as follows (Figure 8):

A restraint and load were then defined. The restraint was placed on the lower surface of the insert. The load was applied to the upper surface of the sled. The model was loaded with a force of 2000 [N], which was distributed over the surface as pressure. The pressure is distributed in units of force, per unit area. The area of the sled to which the pressure was applied is 828 mm^2^. To calculate the pressure, the force of 2000 N is divided by the surface area of 828 mm^2^, which gives a value of 2415 N/mm^2^.

In order to avoid instability, the degrees of freedom of the model were taken away to reflect real conditions. Thus, all degrees of freedom were taken away except for the “Y-Translation”.

The next step was to define the contact pair, element group and materials. The contact pair is the lower surface of the sled and the upper surface of the insert.

The materials used to create the endoprosthesis models are as follows:

CoCrMo alloy—sled,

UHMWPE polyethylene—insert,

Al_2_O_3_ ceramic—insert.

**Figure 9:**
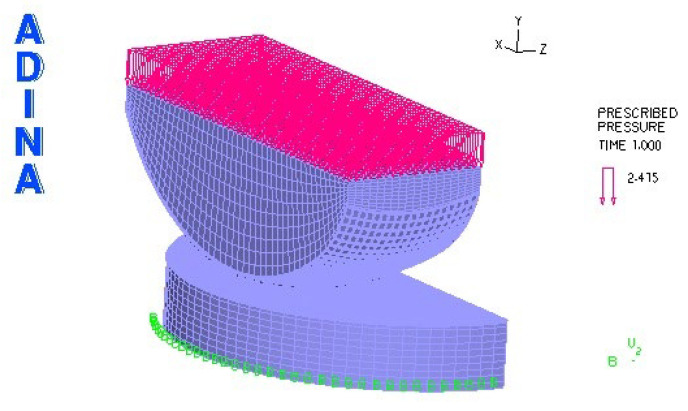
Discrete model view of the modeled solution of knee joint endoprosthesis.

The initial conditions adopted in the numerical problems are illustrated in Figure 1 and Figure 9. Figure 9 illustrates the discrete model view.

## 3. Results and Discussion

### 3.1. Polyethylene Cup

**Figure 10:**
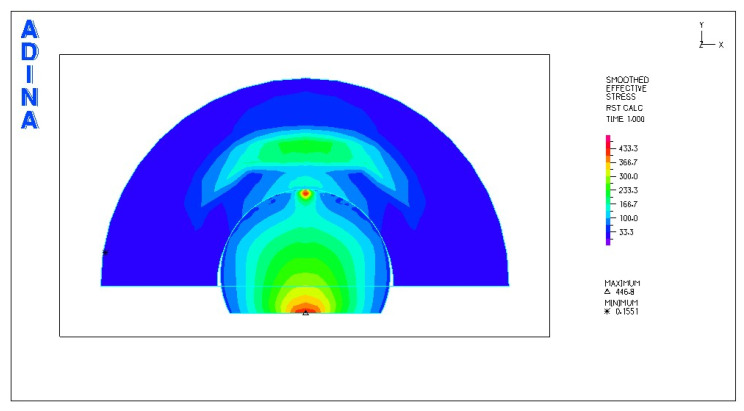
Stress distribution σ_zr_ MPa in the cross-section of the analyzed system for the CoCrMo head–UHMWPE bearing shell connection at a load of 600 N.

**Figure 11:**
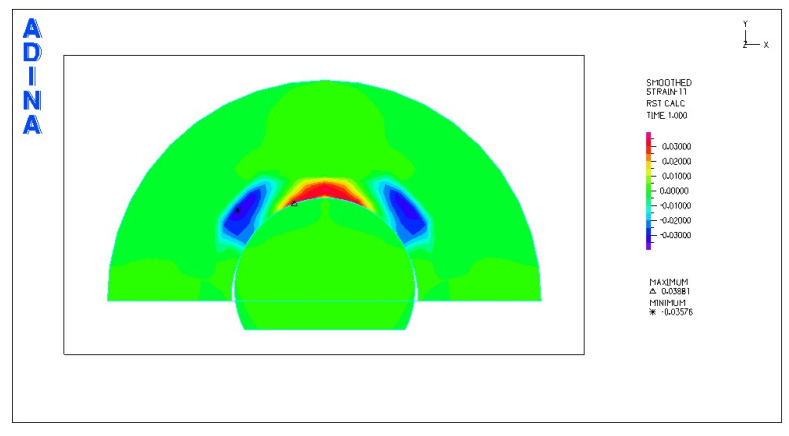
Distribution of strains in the cross-section of the analyzed hip joint endoprosthesis for the CoCrMo head–UHMWPE bearing shell combination at a load of 600 N.

Figure 10 and Figure 11 illustrate sample stress and strain distributions for the analyzed connections.

**Figure 12:**
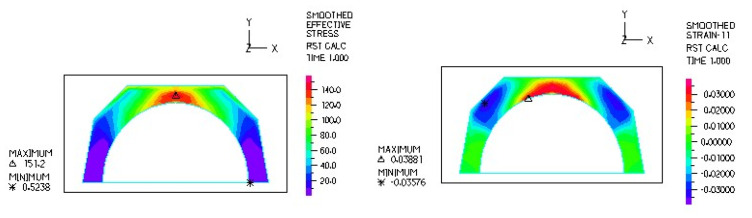
Distribution of strains in the cross-section of the analyzed cup of hip joint endoprosthesis for the CoCrMo head–UHMWPE bearing shell combination at a load of 600 N.

**Figure 13:**
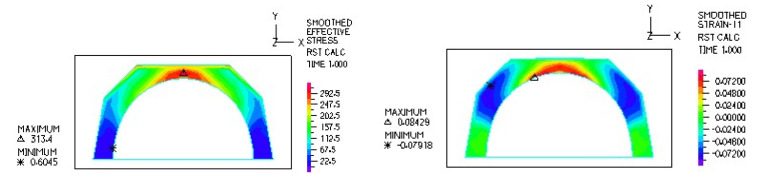
Stress and strain distribution in the bearing shell cross-sections for the CoCrMo head–UHMWPE bearing shell combination at a load of 1500 N.

Figure 12 and Figure 13 illustrate the stress and strain distributions in the UHMWPE-bearing shell for the analyzed association.

**Figure 14:**
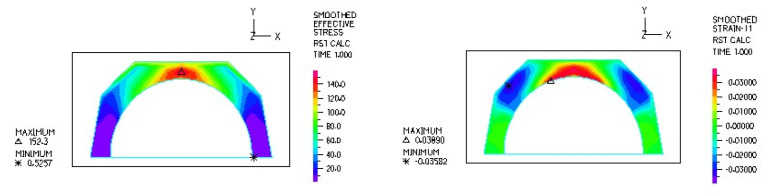
Stress and strain distribution in the bearing shell cross-sections for the ceramic head–UHMWPE bearing shell combination at a load of 600 N.

**Figure 15:**
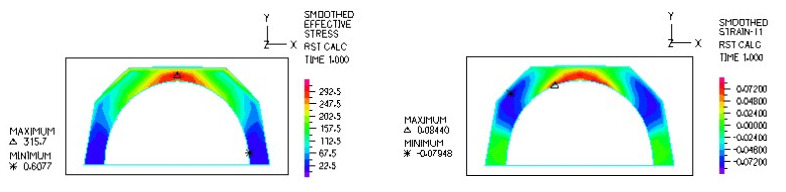
Stress and strain distribution in the bearing shell cross-sections for the ceramic head–UHMWPE bearing shell combination at a load of 1500 N.

Figure 14 and Figure 15 illustrate the stress and strain distributions in the UHMWPE-bearing shell for the analyzed association.

**Figure 16:**
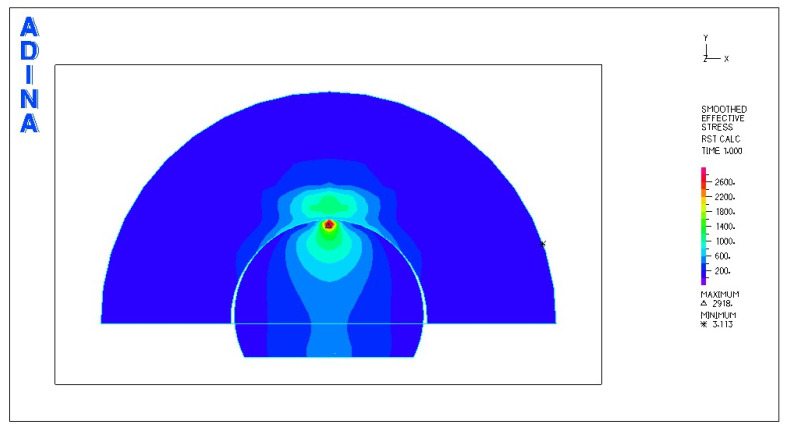
Stress distribution σ_zr_ MPa in the cross-section of the analyzed system for the ceramic head–ceramic bearing shell connection at a load of 600 N.

**Figure 17:**
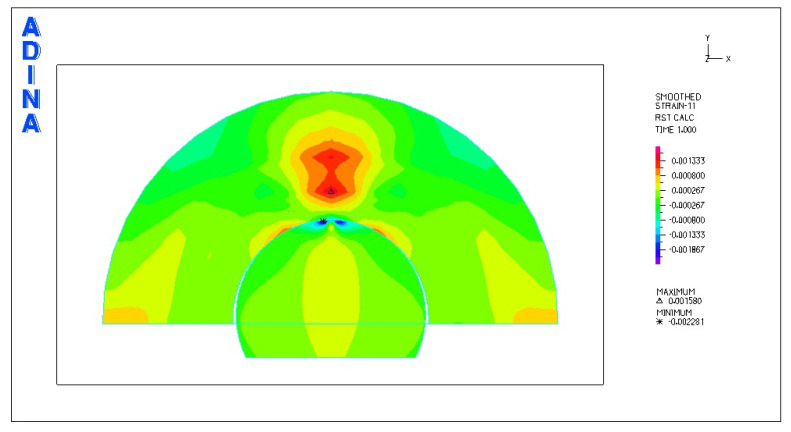
Distribution of strains in the cross-section of the analyzed system for the ceramic head–ceramic bearing shell combination at a load of 600 N.

Figure 16 and Figure 17 illustrate sample stress and strain distributions for the analyzed connections.

**Figure 18:**
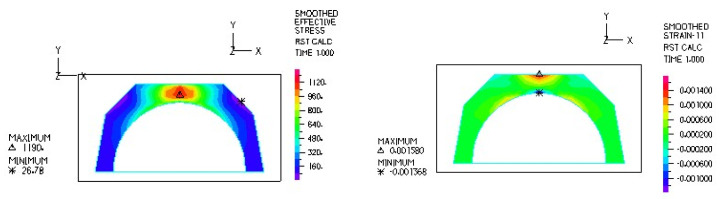
Stress and strain distribution in the bearing shell cross-sections for the ceramic head–ceramic bearing shell combination at a load of 600 N.

**Figure 19:**
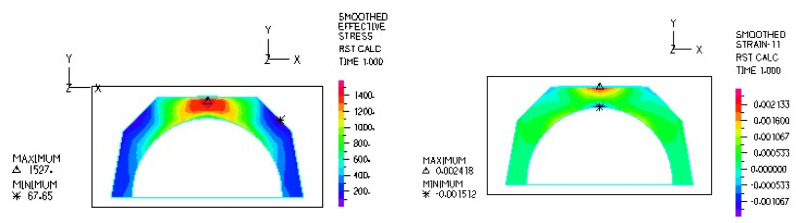
Stress and strain distribution in the bearing shell cross-sections for the ceramic head–ceramic bearing shell combination at a load of 1500 N.

Figure 18 and Figure 19 illustrate the stress and strain distributions in the ceramic bearing shell for the analyzed association.

**Figure 20:**
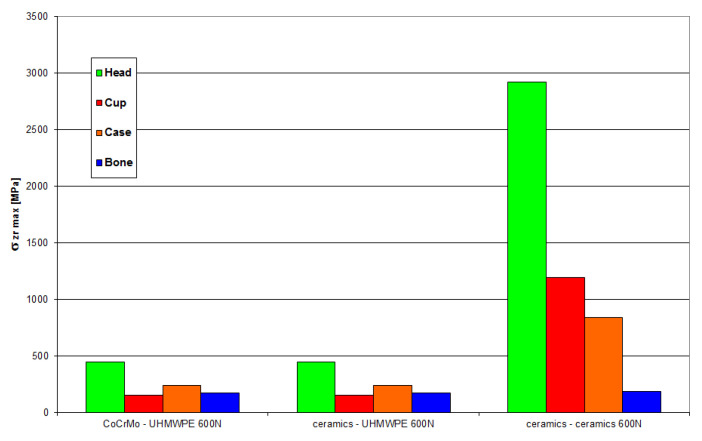
Maximum stress values occurring in individual elements of the “head-cup-case-bone” system at a load of 600 N.

**Figure 21:**
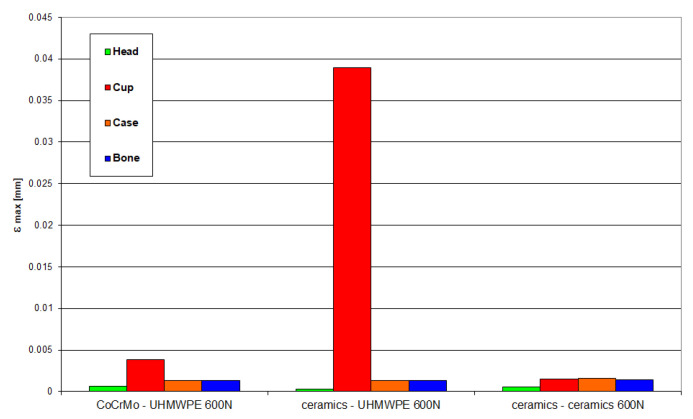
Maximum values of strain occurring in individual elements of the “head-cup-case-bone” system at a load of 600 N.

Figure 20 and Figure 21 present the values of maximum stresses and strains for the analyzed solutions at a load of 600 N.

**Figure 22:**
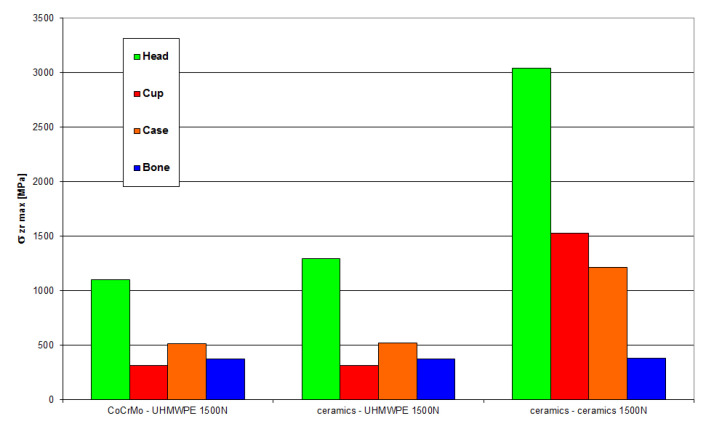
Maximum stress values occurring in individual elements of the “head-cup-case-bone” system at a load of 1500 N.

**Figure 23:**
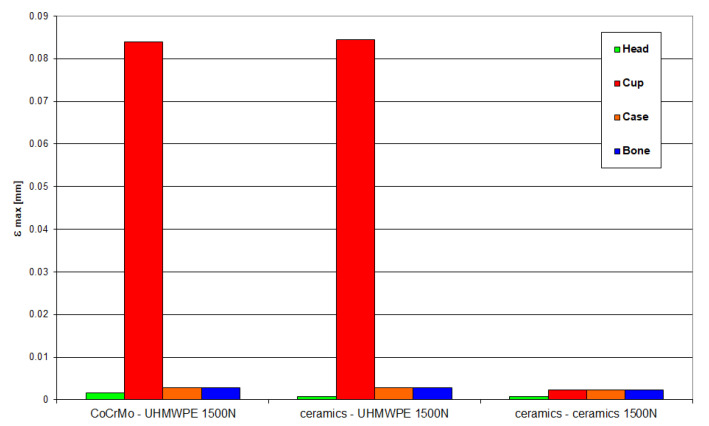
Maximum strain values occurring in individual elements of the “head-cup-case-bone” system at a load of 1500 N.

Figure 22 and Figure 23 present the values of maximum stresses and strains for the analyzed solutions in the case of a load of 1500 N.

Based on the obtained results, it can be stated that the highest value of reduced stresses was obtained for the following combination: head and acetabulum of the endoprosthesis made of bioceramics. At this point, it should be noted that due to the material properties of the cooperating elements, there is a point contact between the cooperating surfaces. At the point of contact, a stress concentration is created, reaching an extremely high value in the subsurface layer, exceeding the compressive strength of the material. It can be concluded that in this place there will be rapid damage of the cooperating elements. Analysis of the stresses of the system indicates a concentration of stresses inside the head of the endoprosthesis. Through the contact between the cooperating surfaces, the stresses are transferred to the acetabulum.

Much lower stress values were obtained for the following pair: ceramic head–UHMWPE cup. Due to the different types of contact of the cooperating surfaces, a more favorable stress distribution was obtained. The fact that the pressure is distributed over a larger surface area resulted in the transfer of stress to deeper layers, i.e., not only to the cup, but also to the cup housing.

The lowest stress values were obtained for the following pair: head made of CoCrMo alloy–UHMWPE cup. In this case, the stresses are also transferred through the polyethylene cup to the cup housing. This confirms the adopted assumption that the use of a cup made of UHMWPE is the optimal solution in this type of system.

In all cases, the stress concentration occurs in the subsurface layer of the endoprosthesis head or in the zone located directly at the place of application of the force loading the system.

Due to the design solution used, which consisted of placing a replaceable insert in a titanium housing, there was no transfer of stresses and displacements from the endoprosthesis to the pelvic bone.

The greatest deformations were characteristic of the cooperation of the ceramic head with the acetabulum made of UHMWPE. At the greatest load, high deformations of the endoprosthesis acetabulum of the order of 0.084 mm were achieved in the superficial zone. Deformation of deeper layers, i.e., the acetabulum housing, was also observed.

The deformations occurring in the case of the polyethylene acetabulum cooperating with the head made of the CoCrMo alloy also show high values in the superficial zone.

A different distribution of deformations was obtained for the following pair: ceramic head–ceramic acetabulum. The occurrence of the greatest deformations was observed at the point of connection of the acetabulum with the housing and deformation of the pelvic bone.

### 3.2. Polyethylene Insert

The test results are summarized in tables. Each table shows the reduced stress (Effective Stress) and strain along the Y-axis (Strain YY). These magnitudes are shown in the polyethylene insert, as this is the component most likely to fail. Each table shows a force load with a different value. Table 5, Table 6 and Table 7, with a force load of 800, 1200 and 2000 N, additionally show the aforementioned magnitudes in the ceramic insert for models *R27G8 and *R34G27.

Figure 24, Figure 25, Figure 26, Figure 27, Figure 28, Figure 29, Figure 30, Figure 31, Figure 32, Figure 33, Figure 34 and Figure 35 show graphical representations of example results obtained for the polyethylene insert under 800 N and 2000 N force loading. The values are shown in the inserts of model R27G8. In addition, the distribution of the obtained quantities in the YZ and XY planes at the location of the highest stresses and strains is shown.

A sled endoprosthesis model with a simplified sled structure and a flat insert was analyzed. Numerical calculations using the finite element method were made possible by using the ADINA program. This program enabled the analysis of stress and strain in the model, as represented in the endoprosthesis insert. Three models differing in the size of the transverse radius and three insert models differing in thickness were used in the calculations. This gave nine different combinations.

Based on the analysis of the results obtained, it can be concluded that the distribution of stresses in the polyethylene insert is greatest for the sleds with the smallest transverse radius. The highest values of reduced stresses, when loaded with a force of 2000 N, occurred in insert models with the designations R27G8, R27G13 and R27G27, where the transverse radius is the smallest at 27 mm. The maximum stress values are as follows: 62.46 MPa, 62.33 MPa and 62.32 MPa. In contrast, the smallest stress values in the polyethylene insert occurred when the sled with the largest transverse radius of 34 mm was used.

Regarding the thickness of the polyethylene inserts, the stress values were highest in the inserts with the smallest thickness of 8 mm and lowest in the thickest inserts of 27 mm. However, since the differences in stresses between inserts of different thicknesses are negligible, the thickness of the inserts does not fundamentally affect the stress values.

Analysis of the calculations shows that the largest linear strain occurred in the polyethylene inserts using a sled with a larger transverse radius. For the models loaded with a force of 2000 N, the largest strain occurred in model R34G8 and was −0.06533, and the smallest in model R27G27 and was −0.06119. Larger strains can be observed in inserts with a smaller thickness, but these are small differences.

From the above observations, it can be concluded that the transverse radius of the skid has the greatest influence on the stress distribution. The larger the radius and the larger the contact area, the lower the stresses. The optimum stress distribution occurs in endoprostheses with the largest contact area. However, the use of endoprostheses with a larger transverse radius limits the range of mobility in the joint to some extent.

Models *R27G8 and *R34G27 additionally show the distribution of stresses and strains in the ceramic insert. These are the models with extreme geometrical parameters, i.e., the sled with the smallest transverse radius together with the insert with the smallest thickness and the sled with the largest transverse radius together with the insert with the largest thickness. After analyzing the obtained calculation results for these contact pairs, it can be concluded that, with the same parameters as in models R27G8 and R34G27, the reduced stresses in the ceramic insert are significantly higher than in the polyethylene insert. Both models were loaded with a force equal to 2000 N. In the ceramic insert, the maximum stress is 624.4 MPa, while in the polyethylene insert it is 62.46 MPa. However, as far as strain values are concerned, they are significantly lower than those of the polyethylene insert.

As for the thickness of the polyethylene inserts, the stress values were highest for inserts with the smallest thickness of 8 mm and lowest for the thickest inserts of 27 mm. However, since the differences in stress between inserts of different thicknesses are negligible, the thickness of the inserts does not fundamentally affect the stress values.

Although the stress values when using ceramic inserts are ten times higher than those of polyethylene inserts, these values do not cause damage to the ceramic material. The stress values generated in ceramic inserts do not exceed the permissible values for this material. The only disadvantage of ceramic inserts is the lower cushioning compared to polyethylene inserts. The metal–ceramic friction pair has good tribological performance. In addition, ceramics have greater biocompatibility than polyethylene. Hence, it can be concluded that ceramic materials can be used for insoles in knee endoprostheses. Numerical calculations for knee endoprostheses fitted with ceramic inserts should be continued in the future.

## 4. Conclusions

In the adopted system, the distribution of stresses and strains varies depending on the material solution used. Depending on the liner used, polyethylene or ceramic, different levels of stress and strain are achieved.

The cooperation of the acetabulum and the head of the endoprosthesis made of bioceramics is characterized by a point contact, and at this point there is a concentration of stresses, reaching very high values.

In almost all cases, the calculated numerical stress values exceed the allowable stress values for UHMWPE polyethylene, which, depending on the grade, are about 10 N/mm^2^ MPa. In addition, the calculated stress values also exceed the yield stress value for this material of 21.5 N/mm^2^ MPa, but at very high loads carried out by simulator experiments and set for numerical calculations. The loads assumed for numerical analysis per single limb were: 800 N, 1200 N and 2000 N for the knee joint, and 600 N and 1500 N for the hip joint. Under real conditions, the endoprosthesis functions under a lower load, as the weight of the patient after implantation is much lower.

For a metallic material (CoCrMo or Ti6Al4V alloy) with a Poisson number ν = 0.3, we obtain σ_0_ = 0.2 σ_max_, that is, the reduced stress occurring at the Hertz point is only 20% of the nominal stress. From the obtained relationship, it is clear that the stress σ_max_ without harming the durability of the polyethylene insert can be much higher than the stress allowed in ordinary tension or compression.

As for the obtained stress–strain distribution for ceramic elements in endoprostheses under the analyzed loading system, the mentioned elements will not be damaged. All the results for the ceramic elements were positively confirmed in empirical studies conducted on joint simulators by the authors. After loading the actual “ceramic head-ceramic acetabulum” set with a force of 600 N and 1500 N, respectively, the friction coefficient values for this set run increased significantly. However, this did not cause damage to the ceramic components analyzed. The highest values of deformation arose at the point of contact between the acetabulum and the head of the hip endoprosthesis.

In knee endoprostheses, conversely, despite the fact that the stress values for ceramic inserts are many times higher than for polyethylene inserts, these values do not cause damage to the ceramic material. The generated stress values of ceramic inserts do not exceed the permissible values for this material.

The maximum stress values obtained exceeded the compressive strength of polyethylene UHMWPE.

When polyethylene acetabularies cooperate with heads made of bioceramics or CoCrMo alloy, there is a high loading of the acetabular material, causing large deformations. There is a transfer of stresses to the layers located deeper, i.e., to the housing and, what is extremely unfavorable, to the bone tissue surrounding the implant.

Such high loading of polyethylene inserts leads to large deformations, which can even cause their destruction.

It can be deduced that the distribution of stress is most influenced by the transverse radius of the skids. The larger the radius and the larger the contact area, the lower the stress values generated in the inserts. The optimal stress distribution occurs in endoprostheses with the largest contact area.

The smallest reduced stresses were obtained for the design variant R34G27-41.32 MPa, with a load of 800 N, where the radius of the cross-section of the skid was 34 mm. The highest stresses occurred for model R27G8-46.20 MPa, where the radius of the skid was the smallest at 27 mm. In the case of models loaded with a force of 2000 N, the largest deformations occurred in model R34G8 and amounted to −0.06533, and the smallest occurred in model R27G27 and amounted to −0.06119. Larger deformations can be observed in inserts with a smaller thickness, but these are small differences.

Finite element calculations do not represent an exact analysis of the object under study, and the results may differ to some extent from the actual values. The accuracy of the results obtained depends on the mesh density of the model and the material data adopted. In the models discussed in this paper, an eight-node mesh was used. Using a mesh with this number of nodes does not give precise results, but using a mesh with a larger number of nodes would significantly increase the calculation time. The number of nodes used in this study is sufficient to perform stress and strain analysis.

The finite element method allows various types of strength simulations and computational analyses to be carried out in the design process on virtual (close to real) models, eliminating the need for geometrically different endoprosthesis prototypes, which require the commitment of a lot of money, time and wear tests on test benches. Thanks to the results obtained by numerical calculations, it is possible to easily and quickly, on the basis of maps of the distribution of stresses and deformations occurring in the elements of the endoprosthesis, propose an optimized geometry of the implant, taking into account the value of the transverse and longitudinal cross-sectional radius of the knee endoprosthesis skids or the thickness and shape of the polyethylene insert. By selecting these quantities optimally, it is possible to ensure that the pressure generated in the polyethylene insert by the skid is low while maintaining mobility in the implanted knee joint. In contrast, material selection with the established optimal geometry of the endoprosthesis is a matter that is limited to easily and quickly changing material parameters in the computer program, such as Young’s modulus, Poisson’s ratio or strength properties of the individual materials used in the simulation.

When designing a model of a sled endoprosthesis of the knee joint, one should be guided by the fact that the transverse radius of the skid should be as large as possible, as this gives the smallest stress distribution. It is also important to bear in mind that the larger the radius, the less mobility there is in the joint. Conversely, a smaller transverse radius provides greater mobility but generates greater stresses.

Numerical calculations for knee endoprostheses fitted with ceramic inserts should be continued in the future.

## Figures and Tables

**Figure 24:**
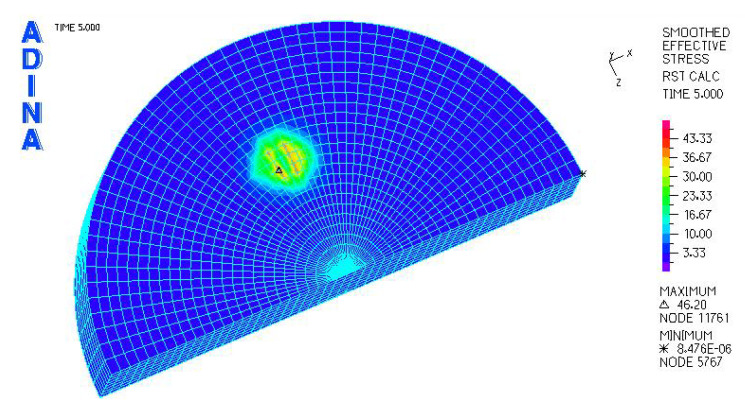
R27G8 model—reduced stress distribution, load force 800 N.

**Figure 25:**
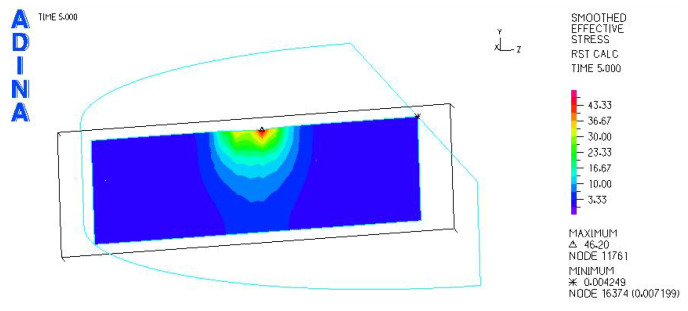
R27G8 model—reduced stress distribution (YZ plane), load force 800 N.

**Figure 26:**
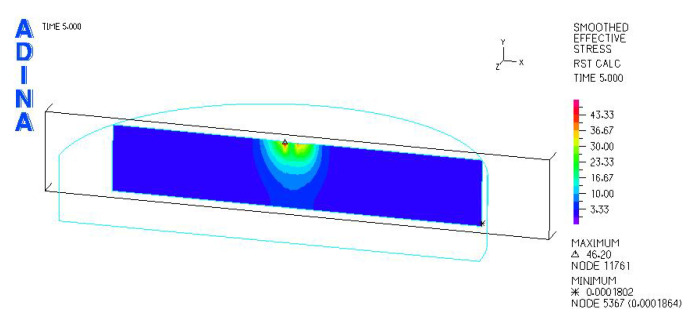
R27G8 model—reduced stress distribution (XY plane), load force 800 N.

**Figure 27:**
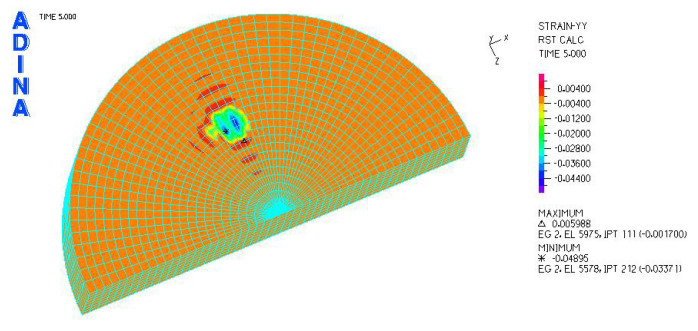
R27G8 model—linear strain distribution, load force 800 N.

**Figure 28:**
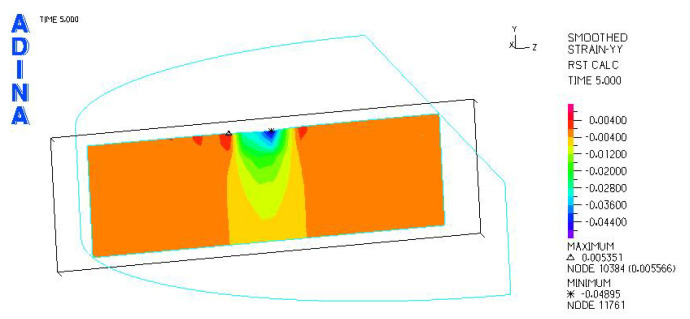
R27G8 model—distribution of linear strain along the Y axis (YZ plane), load force 800 N.

**Figure 29:**
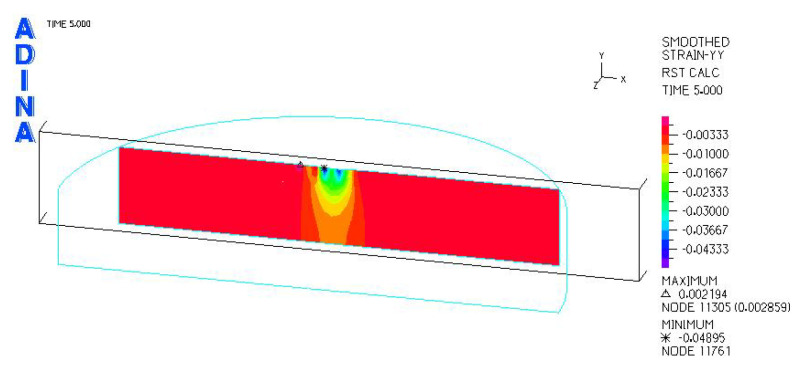
R27G8 model—distribution of linear strain along the Y axis (XY plane), load force 800 N.

**Figure 30:**
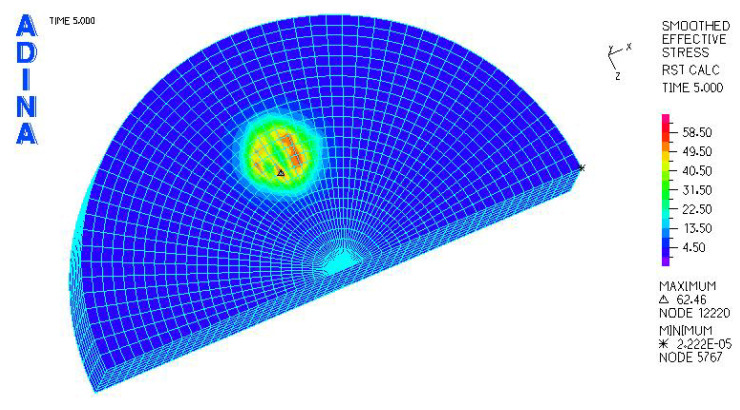
R27G8 model—reduced stress distribution, load force 2000 N.

**Figure 31:**
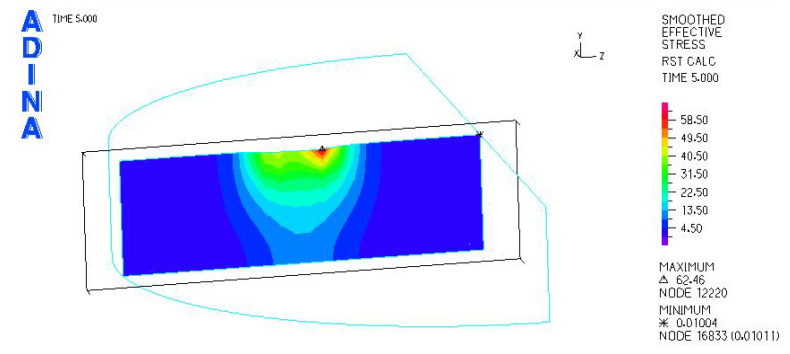
R27G8 model—distribution of reduced stresses along the Y axis (YZ plane), load force 2000 N.

**Figure 32:**
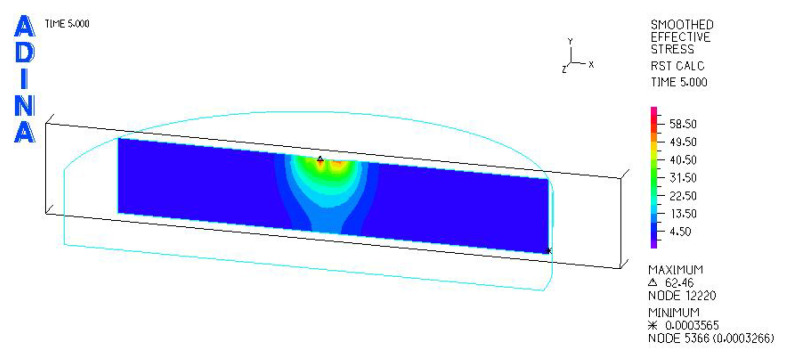
R27G8 model—distribution of reduced stresses along the Y axis (XY plane), load force 2000 N.

**Figure 33:**
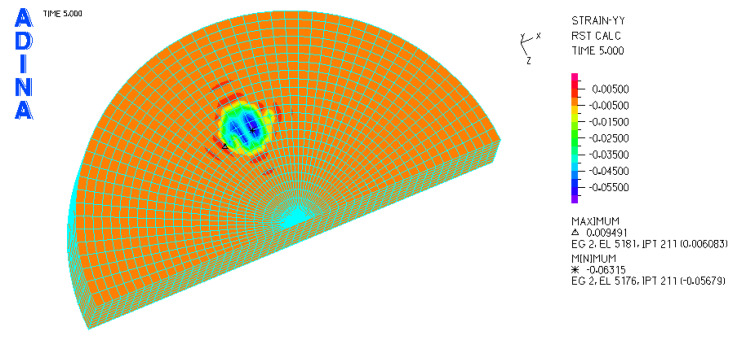
R27G8 model—linear strain distribution, load force 2000 N.

**Figure 34:**
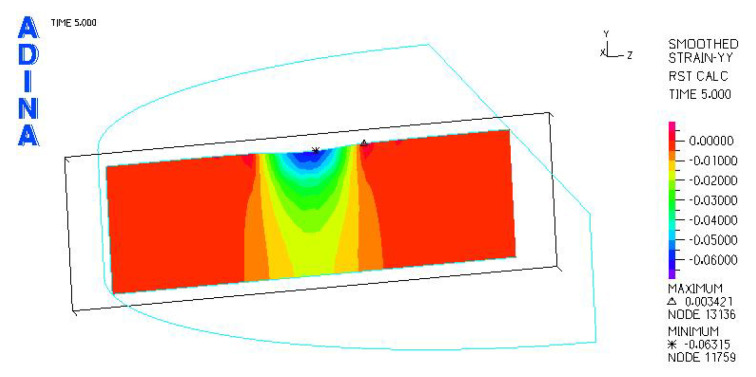
R27G8 model—distribution of linear strain along the Y axis (YZ plane), load force 2000 N.

**Figure 35:**
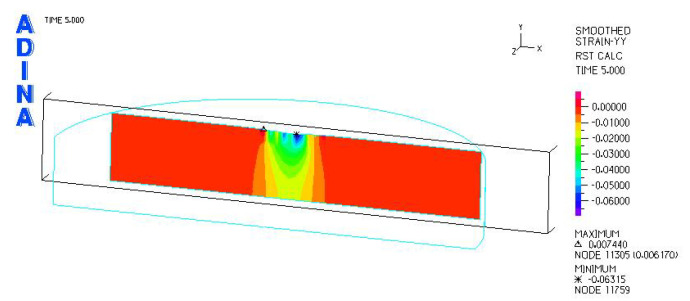
R27G8 model—distribution of linear strain along the Y axis (XY plane), load force 2000 N.

**Table 1:** Mechanical properties of biomaterials and bone tissue [2].

Model Element	Young’s Modulus [MPa]	Poisson’s Ratio ν
cortical bone	1.7 × 10^4^	0.35
CoCrMo alloy	2.0 × 10^5^	0.3
Ti6Al4V alloy	1.1 × 10^5^	0.3
UHMWPE	1.0 × 10^3^	0.4
Al_2_O_3_ ceramics	3.8 × 10^5^	0.22

**Table 2:** Geometrical dimensions of the sleds mm.

	R1	R2	R3	L	g	a
Sled 1	15	28	**27**	46	18	8
Sled 2	15	28	**30**	46	18	8
Sled 3	15	28	**34**	46	18	8

**Table 3:** Geometrical dimensions of inserts mm.

	G	L	r
Insert 1	**8**	50	25
Insert 2	**13**	50	25
Insert 3	**27**	50	25

**Table 4:** Designations of the models analyzed.

		Insert G		
		8	13	27
Sled R	27	R27G8	R27G13	R27G27
	30	R30G8	R30G13	R30G27
	34	R34G8	R34G13	R34G27

**Table 5:** Calculation results with a force load of 800 N.

		Reduced Stress [MPa]		Strain [−]	
		Max	Min	Max	Min
Model	R27G8	46.20	0.000008476	0.005988	−0.04895
	R27G13	45.93	0.0005336	0.006060	−0.04855
	R27G27	45.85	0.007714	0.006087	−0.04844
	R30G8	45.33	0.000008497	0.005998	−0.04831
	R30G13	44.78	0.0005344	0.006059	−0.04751
	R30G27	44.60	0.007721	0.006064	−0.04727
	R34G8	41.98	0.000008504	0.003458	−0.04410
	R34G13	41.41	0.0005350	0.003208	−0.04329
	R34G27	41.32	0.007722	0.003174	−0.04312

**Table 6:** Calculation results with a force load of 1200 N.

		Reduced Stress [MPa]		Strain [−]	
		Max	Min	Max	Min
Model	R27G8	49.70	0.00001261	0.005446	−0.05405
	R27G13	48.93	0.0007901	0.005707	−0.05294
	R27G27	48.68	0.01145	0.005780	−0.05258
	R30G8	46.60	0.00001261	0.006415	−0.50860
	R30G13	45.85	0.0007900	0.006686	−0.04997
	R30G27	45.67	0.01145	0.006745	−0.04968
	R34G8	44.91	0.00001260	0.007441	−0.04911
	R34G13	44.86	0.0007903	0.007661	−0.04819
	R34G27	44.83	0.01145	0.007767	−0.04794

**Table 7:** Calculation results with a force load of 2000 N.

		Reduced Stress [MPa]		Strain [−]	
		Max	Min	Max	Min
Model	R27G8	62.46	0.00002222	0.009491	−0.06315
	R27G13	62.33	0.001352	0.009681	−0.06168
	R27G27	62.32	0.01924	0.009770	−0.06119
	R30G8	62.05	0.00002215	0.009181	−0.06306
	R30G13	61.88	0.001349	0.009366	−0.06262
	R30G27	61.84	0.01922	0.009460	−0.06260
	R34G8	61.79	0.00002207	0.008859	−0.06533
	R34G13	61.71	0.001346	0.009055	−0.06508
	R34G27	61.65	0.01919	0.009117	−0.06475

## Data Availability

The original contributions presented in the study are included in the article; further inquiries can be directed to the corresponding author.
